# Vector-Focused Approaches to Curb Malaria Transmission in the Brazilian Amazon: An Overview of Current and Future Challenges and Strategies

**DOI:** 10.3390/tropicalmed5040161

**Published:** 2020-10-20

**Authors:** Elerson Matos Rocha, Ricardo de Melo Katak, Juan Campos de Oliveira, Maisa da Silva Araujo, Bianca Cechetto Carlos, Roberto Galizi, Frederic Tripet, Osvaldo Marinotti, Jayme A. Souza-Neto

**Affiliations:** 1 Programa de Pós-Graduação em Biotecnologia, Universidade Federal do Amazonas—PPGBIOTEC/UFAM, Manaus 69067-005, Brazil; elerson.matos@hotmail.com (E.M.R.); ricardokatak@hotmail.com (R.d.M.K.); juanbio1807@gmail.com (J.C.d.O.); 2 Laboratory of Medical Entomology, Oswaldo Cruz Foundation, FIOCRUZ RONDONIA, Porto Velho, RO 76812-245, Brazil; maisaraujo@gmail.com; 3 Department of Bioprocesses and Biotechnology, School of Agricultural Sciences, São Paulo State University (UNESP), Botucatu 18610-034, Brazil; bc.carlos@unesp.br; 4 Central Multiuser Laboratory, School of Agricultural Sciences, São Paulo State University (UNESP), Botucatu 18610-034, Brazil; 5 Centre of Applied Entomology and Parasitology, School of Life Sciences, Keele University, Staffordshire ST5 5GB, UK; r.galizi@keele.ac.uk (R.G.); f.tripet@keele.ac.uk (F.T.); 6 MTEKPrime, Aliso Viejo, CA 92656, USA

**Keywords:** malaria, Amazon, Brazil, *Anopheles darlingi*, *Plasmodium*, control, challenges, strategies, conventional, novel, vector, mosquito

## Abstract

In Brazil, malaria transmission is mostly confined to the Amazon, where substantial progress has been made towards disease control in the past decade. Vector control has been historically considered a fundamental part of the main malaria control programs implemented in Brazil. However, the conventional vector-control tools have been insufficient to control or eliminate local vector populations due to the complexity of the Amazonian rainforest environment and ecological features of malaria vector species in the Amazon, especially *Anopheles darlingi*. Malaria elimination in Brazil and worldwide eradication will require a combination of conventional and new approaches that takes into account the regional specificities of vector populations and malaria transmission dynamics. Here we present an overview on both conventional and novel promising vector-focused tools to curb malaria transmission in the Brazilian Amazon. If well designed and employed, vector-based approaches may improve the implementation of malaria-control programs, particularly in remote or difficult-to-access areas and in regions where existing interventions have been unable to eliminate disease transmission. However, much effort still has to be put into research expanding the knowledge of neotropical malaria vectors to set the steppingstones for the optimization of conventional and development of innovative vector-control tools.

## 1. Introduction

Malaria eradication, defined as the permanent reduction to zero of the worldwide incidence of malaria infection, has been a major global and public health objective for decades. Progress toward eradication includes efforts for controlling and eventually eliminating malaria in specific geographic areas or countries. While control measures aim at reducing the number of new infections and the number of people infected in local settings, malaria elimination is accomplished when transmission ceases completely to occur locally. To achieve malaria elimination in Brazil and worldwide eradication, a combination of conventional and new approaches and tools will be necessary [1,2].

In 2015, the World Health Organization (WHO) adopted The Global Technical Strategy for Malaria 2016–2030, providing guidance to countries in their efforts to achieve malaria elimination and setting a goal of reducing global malaria incidence and mortality rates by at least 90% by 2030 [3,4]. The Pan American Health Organization (PAHO) followed with the resolution CD55.R7, a plan of action for malaria elimination in the Americas [5]. In the same year, the Brazilian National Malaria Control Program (NMCP) of the Ministry of Health launched a plan for elimination of the malaria-causing parasite *Plasmodium falciparum* in Brazil [6], acknowledging that *Plasmodium vivax* elimination is more challenging and may take longer, requiring specific tools and strategies for its containment, especially regarding the prevention of relapses [7].

Malaria is a vector-borne disease transmitted by anopheline mosquitoes [8]. Hence, vector control is a vital component of malaria prevention, control and elimination strategies [8,9]. Here we review malaria control measures focused on mosquito vectors presently applied in the Brazilian Amazon and discuss their advantages and limitations. Furthermore, we discuss progresses in innovative vector control approaches aimed at curbing malaria transmission, which once fully developed may be incorporated into integrated mosquito management programs.

## 2. Malaria in Brazil, a Brief History and Current Status

In Brazil, malaria affecting members of the native Tupinambá people was first reported in 1587, however, no epidemics were reported during the colonial period [10,11]. By the end of the 19th century and beginning of the 20th century, in a changed scenario, malaria was endemic throughout the country, with approximately 6 million cases per year [10]. Since then, the disease has virtually been eliminated in the southern areas, where nowadays only a few cases of autochthonous malaria transmission are reported annually [12,13]. In contrast, malaria remains a major public health problem in Northern Brazil, mostly in the Amazon region where more than 99% of the country’s malaria cases currently occur [14,15]. 

The first noticeable increase in the number of malaria cases in the Amazon region occurred during the Amazon Rubber Boom (1879 to 1912), when approximately half a million immigrants moved to the area attracted by job opportunities in latex extraction, natural rubber industrial processing and related activities. Railroads were built to facilitate the transport and export of rubber products and improve accessibility to settled but isolated areas. One of these, the Madeira Mamoré railway, built between 1907 and 1912, was nicknamed the “Devil Railway” because thousands of workers died during its construction [10], largely due to the high number of mosquitoes spreading malaria in the settlements [16]. Just a few decades later, in the 1930s, *Anopheles arabiensis*, coming from Africa by sea, was introduced in Brazil [17,18]. The spread of this efficient vector throughout Northeastern Brazil resulted in an epidemic with more than 150,000 cases and 14,000 deaths from malaria between 1938 and 1939. Fortunately, the invasive *An. arabiensis* population was eliminated in 1940, thanks to management of breeding sites and insecticides sprayed in homes and vehicles [19,20,21].

Despite the elimination of *An. arabiensis* in the early 1940s, malaria continued to be transmitted in the Amazonian states by local anopheline vectors, with estimates of six to eight million people infected and 80,000 deaths annually during that decade [13]. In 1947, the Brazilian National Malaria Service implemented the use of dichlorodiphenyltrichloroethane (DDT) for vector control, and in 1950 of chloroquine for the treatment of patients infected with parasites [22,23]. These measures resulted in significant reduction of malaria transmission, with only 36,900 cases reported in 1961 [19,22]. The spread of malaria in the Brazilian Amazon increased again in the 60’s with the building of new roads, followed in the 70’s with the establishment of hydroelectric projects and in the 80’s with the emergence of the gold prospecting sites [24,25].

In the early 1970s, another immigratory flow was triggered by land ownership opportunities in the Amazon region, when the National Institute for Colonization and Agrarian Reform - INCRA was donating plots nearby the new road network [26,27]. These immigrants, unprepared and unaware, entered a region infested by mosquitoes, resulting in an epidemic with numbers that reached 300,000 cases in a population of 1 million inhabitants in Rondônia state alone [19,25,26,28]. In addition to the migratory wave, between 1970 and 1980, the increase in malaria cases in the Amazonian region was fueled by cuts in funding to sustain the social sectors, including vector control programs [29].

**Figure 1:**
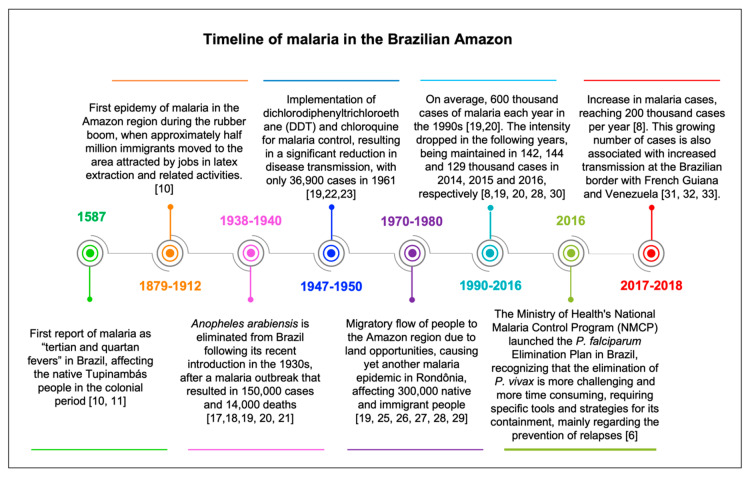
Timeline of the main events concerning malaria transmission and control in the Brazilian Amazon.

From 1990 to 2006, an average of 600,000 malaria cases were recorded annually [19,20], however, intensification of vector control and other malaria prevention and treatment measures in the following years resulted in a steady and significant reduction in the incidence of malaria [28,30]. Transmission intensity was maintained at 140,000 cases yearly in 2014, 2015 and 2016. However, in 2017 and 2018, 197,000 and 207,000 cases were registered respectively, a considerable growth compared to previous years [8]. The recent increase in reported malaria cases is mostly due to transmission occurring near the borders between Brazil and its neighbors French Guiana and Venezuela [31,32,33]. A timeline of malaria in the Brazilian Amazon is presented in Figure 1.

## 3. Malaria Vectors in the Brazilian Amazon and the Importance of Vector Population Surveillance

**Figure 2:**
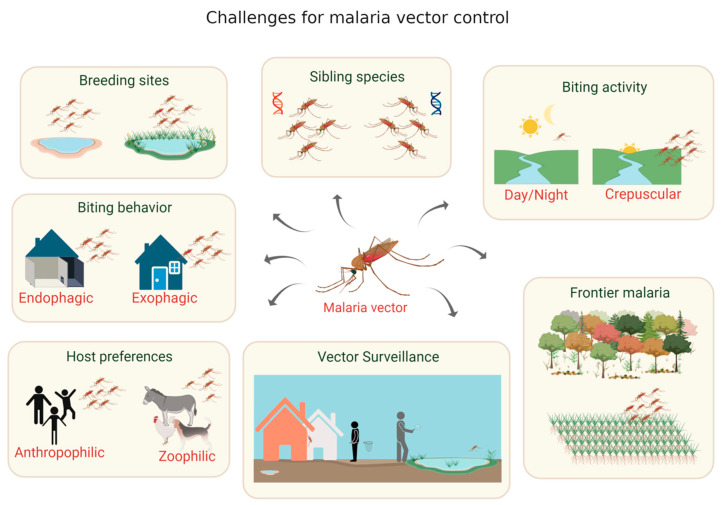
Main challenges for the development of novel vector-focused malaria control tools. Created with BioRender.com.

Effective vector control in the Amazon is a complex and multifactorial task because of the sheer geographical scale of the region, its markedly heterogeneous ecology and complex human demographic aspects [34]. Urban, peri-urban and rural environments, as well as other areas of special interest, as described below, present their unique challenges for controlling malaria transmission. Mosquito density and ecology as well as environmental conditions and human activity must be considered when designing vector-control measures adapted to regional specificities (Figure 2).

For instance, malaria control in areas of special interest such as native people settlements, areas of mining and national borders, present their own challenges. Malaria incidence in Brazilian native people is associated with environmental changes, their difficulty in accessing health services, and their mobility, at times resulting in migration to areas of more intense malaria transmission [35,36]. Furthermore, culturally determined activities such as hunting, fishing, working in the fields, and bathing along rivers and streams expose indigenous people to the risk of malaria infection. Gold mining plays a major role in spreading malaria in the area [37] since this activity creates puddles of water, ideal habitat for the reproduction of *Anopheles* mosquitoes. Transmission of malaria among miners (garimpeiros) is greatly affected by their constant mobility and a work regime that coincides with the peak of biting activity of vector mosquitoes. Populations of border towns are generally more vulnerable, especially those living in remote areas, as recently observed along the border between French Guiana and Brazil where local inhabitants were affected by a malaria outbreak [38]. Recent events in Venezuela, causing mass migrations, have been responsible for an increase in malaria transmission across the frontier between Brazil and Venezuela [31,32]. Each one of these scenarios demand unique malaria control programs that include vector-control measures adapted to the regional context.

**Figure 3:**
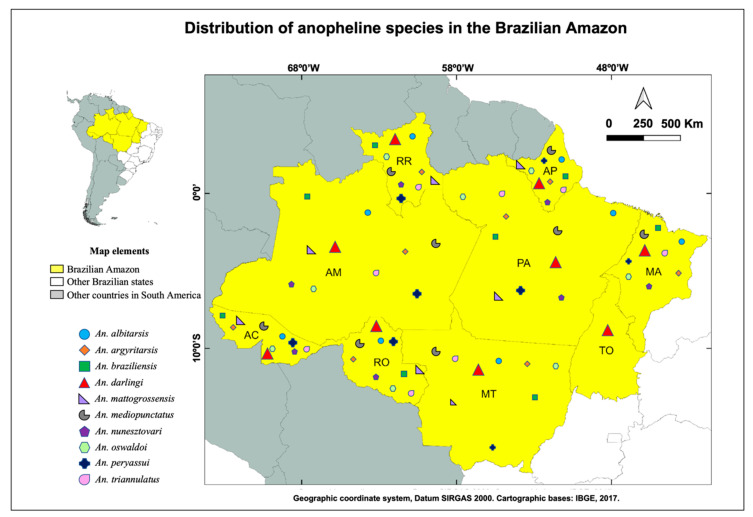
Distribution of the main anopheline species known to occur in the Brazilian Amazon, with emphasis in *Anopheles darlingi*, the main malaria vector in the region. The mosquito species distribution was carried out according to a database previously published by [28].

A total of fifty-four species of Anopheles are known to occur in Brazil, 33 of which are found in the Amazon region [39,40]. *Anopheles darlingi*, *An. albitarsis*, *An. braziliensis*, *An. nunesztovari*, *An. oswaldoi*, *An. triannulatus*, *An. mattogrossensis*, *An. mediopunctatus* and *An. peryassui* among other anopheline species are found throughout the Amazonian region [28] (Figure 3). All of those listed above are susceptible to natural *Plasmodium infection*, as demonstrated by using ELISA detection of the circumsporozoite protein (CSP), microscopy and/or PCR [28,41]. Among those, the primary vector in the Amazonian Rainforest malaria transmission system is *An. darlingi* [28], which is abundant and highly anthropophilic [42,43]. *Anopheles darlingi* readily adapts to environmental changes caused by human activity and can easily develop in either artificial breeding sites such as fish farm tanks or in the midst of nature [44,45]. While one must recognize the economic importance of fish farming in the Brazilian Amazon region, its contribution to increased vector populations and malaria transmission is clear. Active and abandoned fishponds with shaded, deep, clean waters and surrounded by vegetation without consistent cleaning of their borders, may form suitable breeding sites for *An. darlingi* and other anopheline mosquitoes [46].

Vector surveillance is essential to inform vector control strategies and evaluate their impact on malaria transmission. In the Amazon, vector surveillance has evidenced geographical and temporal differences in mosquito densities and species composition in malaria endemic areas [39,47]. Factors driving this diversity are environmental [48,49], anthropogenic [45] and biological [39,50]. Hydrological cycles of the Amazon region include heavy rainfall between the months of November and June, resulting in the flooding of approximately 85,000 km^2^ of the Amazonian plain [51]. The end of each flooding cycle creates numerous large and small pools of water and slow-flowing streams suitable for mosquito breeding, as water levels slowly recede along rivers [45]. The large-scale climate interaction caused by environmental phenomena like the El Niño are associated with warmer temperatures, higher dew points, as well as reduced precipitation and river discharge in the Amazon, which may influence the dynamics of malaria transmission [52,53].

Anthropic activity in the Amazon has been associated with “Frontier malaria” a term commonly used to describe malaria transmission related with deforestation and with the unplanned development of new agricultural settlements [54]. Newly deforested areas create multiple new *An. darlingi* breeding sites, favoring malaria transmission [55,56]. However, anthropic activity may favor other vector species too. In Macapa, such changes reduced the suitability of breeding sites for *An. darlingi* and led to an increased density of *An. marajoara* [57].

Phenotypic plasticity, including features such as anthropophily and endo-exophily, have been revealed by entomological surveys of neotropical malaria vectors [58,59]. *Anopheles darlingi* typically displays a single peak of biting activity before midnight [60,61,62,63,64,65], however, in certain areas two peaks of biting activity are observed: one at dusk and another at dawn [66,67,68,69,70]. Furthermore, *An. darlingi* biting activity may occur in three peaks at sunset, midnight and dawn [71]. Observations at one locality showed considerable plasticity of this species’ biting patterns as well, indicating that intra-population variation of biting activity can be as significant as inter-population variation [72]. Single or multiple blood meals in each gonotrophic cycle, determined by biological or environmental factors, also may influence the vectorial capacity of malaria mosquitoes [73].

The investigation of cryptic species among neotropical malaria vectors is essential for a better understanding of species distributions, behavior and population dynamics, leading to a better understanding of malaria transmission and adequate strategies for effective vector control in the Amazon. In Africa and in Asia, several of the major malaria vectors belong to species complexes, including cryptic species that differ in host feeding preference, breeding sites, feeding behavior, and role in malaria transmission [74,75,76]. These complexes may include both malaria vectors and non-vector species, which may either occur sympatrically or have distinct geographical distributions. While *An. darlingi*, the major malaria vector in the Brazilian Amazon, is a monotypic species, other neotropical malaria vectors such as *An. nuneztovari, An. albitarsis*, *An. triannulatus* and *An. oswaldoi* are complexes of closely related, morphologically similar species [77]. For instance, *An. konderi* is often mistaken for *An. oswaldoi* [78], however *An. konderi* is present in human impacted or open areas, whereas *An. oswaldoi* is restricted to forested ones [79]. Marrelli et al. (1999) observed that both *An. oswaldoi* and *An. konderi* developed *P. vivax* oocysts in their midgut, but the complete cycle of the parasite, with sporozoites reaching the salivary glands, was only observed in *An. oswaldoi*, suggesting these species differ in vector competence [80]. Therefore, the refinement of taxonomic tools, including molecular taxonomic tools [81,82] is paramount for the knowledge and understanding of the biology of these neotropical species complexes and for the investigation of malaria epidemiology. Furthermore, proper identification of species and knowledge of their ranges, often affected by changes in land use and lately in the climate, is vital for appropriate allocation of vector control resources.

## 4. Conventional Measures for Vector Control and Their Limitations

The core and supplemental interventions for malaria vector control advocated by the World Health Organization and the Brazilian Ministry of Health are very similar as they represent a set of evidence-based guidelines (Table 1). The Brazilian Health Ministry acknowledges that not all areas should be subject to the same malaria control programs and follows a decentralized system in which each municipality adopts different control strategies. Also, it recognizes the existence of areas of special importance that are particularly vulnerable and with limited access to interventions. These include: Indigenous areas/indigenous tribes; gold mining camps; settlement areas; and frontier areas in the northern and western Brazilian Amazon rainforest.

### 4.1. Long-Lasting Insecticidal Nets (LLINs)

The use of LLINs is a highly cost-effective strategy for malaria prevention that has contributed to a significant reduction in disease morbidity and mortality worldwide. Until 2007, the WHO advocated distribution of LLINs only to pregnant women, children, and human immunodeficiency virus (HIV)-positive individuals. Since then, LLINs are recommended by WHO to all individuals at risk in endemic areas [87]. Accordingly, in Brazil, the Ministry of Health recommends the distribution and installation of LLINs, which are intended for personal overnight protection. Currently, LLINs impregnated with pyrethroid have a shelf life of less than 3 years [88,89,90]. Insecticide-treated bed-net incorporating a mixture of the pyrethroid alpha-cypermethrin and pyriproxyfen, an insect growth regulator, has been proposed as an alternative to pyrethroid-only nets. Experimental evaluations showed more than 80% mortality and higher than 90% blood-feeding inhibition in the African malaria vector *An. gambiae* exposed to these LLINs. Furthermore, blood-fed female mosquitoes surviving net exposure suffered 83% reduction in oviposition and 95% reduction in offspring, indicating a potential improvement of malaria vector control when compared to standard pyrethroid-only LLINs [91]. Insecticide-treated nets with other insecticide combinations such as chlorfenapyr and alpha-cypermethrin, have also been proposed as tools for controlling malaria vector mosquitoes [92]. Impregnation of mosquito nets with antimalarial drugs has been suggested as an alternative to mitigate the problem of insecticide resistance [93]. Evidence-based discussion is needed before these novel mosquito nets enter the development pipeline.

Three-hundred-thousand LLINs have been purchased by the Ministry of Health between 2015 and today. Despite the advantages of using LLINs subsidized by the government, data regarding the actual distribution and use of impregnated mosquito nets in this region is scarce [94,95]. Local surveys indicate negative perceptions of LLINs, as they may cause skin irritations and allergies and are not effective in preventing malaria transmission occurring outdoors [96]. Low compliance, net misuse, lack of LLIN replacement program, and local epidemiological factors may curtail the efficacy of impregnated bed nets for malaria control in the Amazon region. 

### 4.2. Indoor Residual Spraying (IRS)

Another core measure for malaria vector control consists of spraying the walls of commercial buildings and residences with insecticides that remain on the applied surfaces [97]. Etofenprox PM 20% is the insecticide used in Brazil for residual spraying for malaria vector control. This product has a residual effect for 4 months requiring three annual applications [88]. In the Amazon, factors such as the operational cost of mobilizing teams to perform insecticide spraying, the difficulty in accessing remote areas at adequate frequency, the variability of dwellings, and variable environmental conditions, may compromise the efficacy of IRS [98]. In fact, few systematic evaluations on the impact these measures have on suppressing anopheline populations and reducing levels of malaria transmission in the Brazilian Amazon have been performed [88,98]. While IRS is applicable and effective in urban and peri-urban environments, issues need to be addressed regarding gold miners who often live in huts without walls, continuously exposed to mosquito bites; unconventional indigenous house architecture that may not favor IRS; and the lack of studies regarding the stability of insecticides applied on unconventional surfaces and under extreme humidity and temperature environmental conditions [99]. Investments in next generation log lasting insecticides and advances in IRS management and application equipment have offered promising results for effective future application of IRS in the Brazilian Amazon [100,101].

### 4.3. Larvae Control

Supplemental interventions based on larval control are effective in reducing vector density and malaria transmission where aquatic habitats of the principal malaria vector(s) are few, fixed and findable, and where its application is both feasible and cost-effective [102,103,104]. Historical interventions, largely based on larval control, including the previously mentioned eradication of *An. arabiensis* from Brazil [105], suggest that this approach may be an important asset in the battle for achieving malaria elimination and eradication.

Early larvicidal interventions involved environmentally damaging measures including the elimination of breeding sites by filling depressions or draining swamps, and the application of toxic diesel or Paris green, impacting all organisms living in ponds, swamps, and other breeding sites. Nowadays, environmentally friendly alternatives are widely available. For example, biolarvicides based on the bacteria *Bacillus thuringiensis israelensis* (Bti) and/or *Lysinibacillus sphaericus*, (syn. *Bacillus sphaericus*, Bs) have been successfully applied for mosquito control in various ecological settings in sub Saharan Africa [106], Europe [107], Asia [108] and South America [109]. Aquatic insect predators and larvivorous fish have also been proposed as mosquito biocontrol agents, however, there is only limited evidence of their impact on disease transmission [110]. Density, diversity and habitat effects on the efficacy of natural mosquito larvae enemies must be considered. For instance, the presence of alternative preys, normally present in the extremely biodiverse Amazonian environment, together with the scarce selectivity of predators may limit the impact of such approaches on mosquito population [111]. 

Larvae control interventions require substantial knowledge of larval ecology due to the effects of weather and physical and biological characteristics of larval habitats on their efficacy. Furthermore, larviciding interventions are labor-intensive and to be effective must cover multiple *Anopheles* larval habitats often dispersed in vast areas [106]. Therefore, the effectiveness of larvicides in the Amazon is limited to urban and peri urban environments, during the dry season and where the number of mosquito breeding sites is limited and easily accessible. Larviciding is not recommendable when and where breeding sites are inaccessible and countless, and in rural and frontier environments. Because of the territorial dimensions of the Amazon and the characteristics of breeding sites, in rural areas this procedure is restricted to the vicinity of inhabited settlements in ranches and farms and fishponds [112]. Recent developments using unmanned aerial vehicles coupled with high-resolution multispectral imagery to locate anopheline breeding sites could contribute to lower costs and improve the coverage of larvae control programs in the Amazonian region [113,114]. 

### 4.4. Personal Protection

Supplementary prophylactic measures against malaria include personal protection such as screens installed in windows and doors, clothing covering exposed parts of the body during biting periods, mosquito repellents, insecticides and air-conditioning [115]. Although highly recommended for travelers entering malaria endemic areas [116], most types of continuous personal protection may be neither reasonable nor affordable for local inhabitants due to economic and cultural issues. The typical climate conditions of the Amazon, with both high temperature and humidity, make wearing long sleeves shirts and pants uncomfortable as a way of mosquito bite protection. Insecticide impregnated clothing is expensive and requires replacement or special treatment to maintain its protective function [117]. Mosquito repellents require continuous reapplication and costs may be prohibitive. In Brazil DEET-based products (100 mL, of 6.7–7.1% DEET) costs on average 15.80 BRL equivalent to US$3.84 [118]. Furthermore, the efficacy of commercially available repellents in protecting people from neotropical anophelines bites is still largely unknown. Local insecticide spraying is only manageable, sustainable and effective in confined environments, and its continuous application harms the environment [119]. Finally, housing improvements including netting and air conditioning are dependent on traditional architecture, windows and doors, as well as access to electricity and sufficient income that allows purchase, installation and maintenance of such home improvements.

A large body of work is now emerging on housing improvement and materials for malaria control. Lethal house lures, insecticide or mosquito repellent-treated furniture and sandals, and materials for temporary shelters, are offering new tools that can be applied to both stable and traditional or temporary housing settings [120,121,122,123,124,125]. These advances, once implemented in the Amazon, could constitute a scalable and low-cost supplement to current malaria control interventions.

## 5. Promising Novel Vector Control Approaches

In view of the present limitations of conventional techniques for vector-focused malaria control in the Amazon, novel and promising approaches to curb malaria transmission are under investigation and consideration. 

### 5.1. Genetic Control of Malaria Vectors

Genetic engineering of mosquitoes offers solutions and novel strategies to tackle the challenges encountered by current vector control interventions such as: difficulties in deploying control measures to the affected regions, largely rural and dispersed in large areas; the broad-spectrum activity of available insecticides and the spread of insecticide resistance. Such strategies rely on the release of modified insects carrying specific genetic traits, which act upon mating with the compatible species. This limits their impact on the ecosystem and, at the same time, facilitates the deployment of the intervention by taking advantage of the dispersal and mate-seeking behavior of the released mosquitoes. 

#### 5.1.1. Population Suppression or Replacement

Genetic control strategies can be aimed either at the “suppression” of the overall number of vectors or to their “replacement” with modified insects that are incapable or refractory to the transmission of the pathogen. Suppression strategies usually exploit the engineering of genetic traits that interfere with the reproductive capacity of insects and/or their fitness. Conversely, genetic modifications for population replacement involve the introduction and expression of exogenous antiparasitic genes or the editing of the genetic components involved in vector-pathogen interactions to block or reduce the parasite development within the vector. Replacement modifications can also be intended to hinder the host-seeking behavior of insects, thereby reducing their vector competence [126,127,128].

#### 5.1.2. Self-Limiting or Sustaining Strategies

The genetic control traits carried by the modified insect can be engineered to achieve different levels of persistence in the population after being released; this may vary from one, up to a virtually unlimited number of generations. Classic sterile insect technique (SIT) relies on repeated inundative releases of radio or chemical-sterilized males, which can suppress the targeted populations by exploiting the single-mating capacity of female mosquitoes [129]. However, poor survival and mating competitiveness of the sterile males released are detrimental for the efficacy of these strategies [130]. 

The availability of genomic sequences and tools for the genetic modification of the mosquito genome allowed the engineering of alternative approaches based on the release of genetically modified male insects carrying a dominant lethal gene (RIDL), with the added benefit of a reduced impact on the fitness of released males compared to the classic methods [131]. With both approaches (SIT and RIDL), the sterile or dominant lethal traits carried by released males are not transmitted to the following generations minimizing long-term impacts and simplifying the risk assessment process leading to field applications. Both these technologies have been successfully applied for suppression of agricultural pests and vectors [131,132,133,134] including the New World screwworm fly *Cochliomyia hominivorax* (cause of myiasis) in several American and African countries [135,136], the malaria vector *Anopheles albimanus* in El Salvador [137], the dengue virus-transmitting *Aedes aegypti* in Brazil [138] and tsetse flies (*Glossina* spp.) carriers of the African trypanosomiasis (sleeping sickness) in the Zanzibar Island Unguja [139] among other examples. However, self-limiting methods, such as SIT and RIDL, require repeated mass releases of the modified insects, challenging their use for the treatment of large or remote geographic regions and/or non-isolated vector populations. 

Beside the dominant sterility phenotype associated with these specific methods, genetic modifications are at best neutral or, in most of the cases, conferring a reduction of fitness to the carriers, resulting in a gradual removal of these traits from the population after release [140]. Approaches to overcome these limitations were theorized in the first half of the 20th century when both threshold-dependent and self-sustaining strategies were initially proposed [129,141]. Threshold-dependent strategies, such as genetic underdominance, involve the introduction of genetic elements able to invade a population only if seeded above a certain frequency, which depend upon the fitness of released insects relative to wildtype [142]. On the other hand, self-spreading technologies such as gene drives (GD) offer the advantage of reducing the size and the frequency of releases necessary to either suppress the targeted population or replace it with insects unable to transmit the parasite [140,143,144].

GD elements can be engineered to bias their own transmission by hijacking the mendelian partition of genetic material during germline formation of the vectors hosting such modifications in their genome. For example, site-specific endonucleases, such as the increasingly popular CRISPR-Cas system, can be inserted into specific genomic sequences to disrupt the function of haplosufficient genes with a role on female development [145,146] or fertility [147]. The same endonuclease, active during the diploid stages of germline formation, is programmed to cut the target site on the homologous chromosome (not containing the CRISPR drive element). The double-strand DNA break stimulates the homology directed repair (HDR) machinery of the germ cell to repair the broken chromosome by using its homologous twin, carrying the GD, as a genetic template. As a result, the GD element is copied (“homed”) to the homologous chromosome and transmitted to the entire progeny, instead of only half, thereby increasing in frequency over generations [140,144]. 

The short life cycle of mosquitoes allows a rapid increment of individuals heterozygous for the GD element (and the associated genetic disruption) in the population, even if released at low frequencies, progressively reducing the number of wildtypes. Mating between GD heterozygous mosquitoes generates individuals without functional copies of the targeted haplosufficient gene, manifesting the disruptive phenotype; e.g., female sterile [147] or intersex XX individuals [145], causing suppression of the population. In a recent work, the same CRISPR-based gene drive element was also linked to a second endonuclease, targeting the X chromosome during male meiosis [148] and to bias transmission of sperm in favor of those carrying the unaffected Y chromosome. As a result, super-mendelian transmission of the GD is accompanied by male-biased progenies, thus presenting the advantage of reducing the fraction of biting females whilst suppressing the population [146]. Similarly, CRISPR-based GD constructs homing in neutral genetic loci were also engineered to spread anti-parasite molecules through caged mosquito populations [149,150].

#### 5.1.3. Challenges, Alternatives and Transfer to Neotropical Species (e.g., *An. darlingi*) 

Besides the technical challenges, such as the selection of genetic resistance to the driving component or to the anti-malarial molecule, consistent research efforts have been focused over the last few years towards the development of new methods to limit or mitigate the spread of gene drive elements. The flexibility and modularity of CRISPR endonucleases prompted a variety of genetic control forms with reduced penetrance as well as the development of novel countermeasures to gene drive spread [151,152,153,154,155]. The rapid progress in the laboratory and the potentials offered by these technologies are progressively shifting challenges towards the assessment of risk and ecological impact, regulation and acceptance prior to field applications [156].

The flexibility offered by the molecular components used for genetic control offers the opportunity to transfer, with relative ease, these technologies to other species, such as *An. darlingi*, albeit the following resources are available: a laboratory-adapted inbred colony for genetic manipulation and testing in the laboratory; annotated genome and, favorable but not essential, a transcriptome for the selection of candidate genes and regulatory sequences for the expression of molecular effectors in the mosquitoes. Ad hoc transcriptomes may be unnecessary in the case orthologous genes may be retrievable from sibling species [157,158,159,160,161]. Successes with *An. darlingi* colonization [162,163,164] and genome sequence and annotation [165] and similar advances with other neotropical anophelines [166,167,168,169] offer optimism that these technologies will soon be transferred to neotropical Amazonian malaria vectors.

The use of genetically engineered mosquitoes has the potential of being effective in controlling vector mosquito and/or disease transmission. However, the coexistence of multiple anopheline malaria vectors in the Amazon may pose a challenge for these novel control approaches. Furthermore, transgenic mosquitoes-based methods still require a great deal of work as research is at the trial stages. Efforts for improving sex separation, better transportation and release protocols are required for field optimization. Potential problems related to late accumulation of deleterious mutations on the transformed mosquito populations used for replacement or adaptation of the targeted parasites to the new populations still need to be addressed.

### 5.2. Microbial-Based Approaches to Control Malaria Transmission and Malaria Vector Populations

#### 5.2.1. Entomopathogenic Organisms

Mosquitocidal microorganisms, including viruses, fungi and bacteria have been investigated as potential ecologically friendly alternatives to chemical insecticides [170,171,172,173]. *Bacillus thuringiensis* var. *israelensis* (*Bti*) and *Lysinibacillus sphaericus* or *Bacillus sphaericus* (Bs) selectively kill mosquito larvae and have been used for decades with high efficacy and safety records [174,175]. However, conventional Bti and Bs have low residual activity requiring repeated applications and increasing the cost of interventions [176,177]. Long-lasting microbial larvicide formulations with sustained release of Bti and Bs active ingredients for up to 6 months are currently commercially available [103,178,179,180]. These new long-lasting larvicidal formulations associated with the use of drones to identify and map mosquito breeding sites, and deliver aerial application of granular or aqueous Bti formulations, may assist in reducing the complex operational challenges that affect mosquito control in the Amazon environments [113,181]. Besides Bti and Bs, microorganisms such as the bacteria *Chromobacterium* sp. Panama [182,183] and the fungi *Beauveria bassiana* [184] and *Metarhizium anisopliae* [185], among others, have demonstrated mosquitocidal activities. The present challenge is to convert these promising observations into products that are ready to be incorporated in mosquito control interventions.

Genetic engineering methods have been proposed to increase the pathogenicity, improve longer-term efficacy, and prevent or delay insect resistance to entomopathogenic microorganisms [185,186]. The addition of *Bti* genes into *Bs* genomes to increase infectivity to mosquitoes [187,188] and the expression of *Bti* and non-*Bti* derived mosquito toxins in readily transformable microorganisms such as *Chlorella desiccate*, *Pichia pastoris* and *Saccharomyces cerevisiae* have been investigated as alternatives to develop new microbial products for mosquito control [189,190,191]. 

#### 5.2.2. Naturally Occurring Symbiotic Microorganisms with Anti-Pathogen Activity 

Mosquito microbiomes modulate insect immunity, and some naturally occurring symbiotic microorganisms are capable of hampering or blocking malaria parasites development within their vectors [192,193,194]. These symbionts have been proposed as agents to render mosquito populations refractory to *Plasmodium* [195]. For example, the *Serratia marcescens* strain Y1 promotes the activation of the insect immune system, resulting in a reduction of the number of developing oocysts after mosquitoes are challenged with an infective blood meal [196]. Similarly, another *S. marcescens* strain isolated from *An. albimanus* impairs *P. vivax* infection in that vector [197]. Enterobacter species isolated from wild mosquito populations in Zambia also show anti-*Plasmodium* activity, likely through the production of reactive oxygen species (ROS) [192,197,198]. Bacteria of the genus *Asaia* induce a basal immunity in *Anopheles* mosquitoes, leading to a decrease in the development of malaria parasites within their vectors [199].

The symbiotic yeast *Wickerhamomyces anomalus* (WaF17.12), isolated from the malaria vector mosquito *Anopheles stephensi*, has shown strong anti-plasmodial activity. Mosquitoes colonized with WaF17.12 developed 65.2% fewer parasites than the control group [200]. More recently, a symbiont microsporidian (*Microsporidia MB*) that colonizes *An. arabiensis* from Kenya, was shown capable of blocking *P. falciparum* development and transmission, providing a new prospect for malaria control [201]. Host-baited traps, odour-baited traps, resting traps, and sugar-baited traps have been proposed as possible ways of delivering these agents to mosquitoes [202].

#### 5.2.3. Paratransgenesis

Paratransgenesis consists in genetically transformed mosquito symbionts such as fungi, viruses or bacteria to disrupt the transmission of vector-borne pathogens [203]. The perspective of applying paratransgenesis for malaria control has driven exciting research with promising results. Recombinant densovirus such as AgDNV, isolated from *An. gambiae*, can be used to infect mosquitoes and drive expression of anti-*Plasmodium* peptides to block parasite transmission or insect-specific toxins to reduce mosquito population density or mosquito lifespan [204]. Fungi carrying effector genes that hinders *Plasmodium* development have been genetically engineered [205]. Among the symbiotic bacteria found in malaria vectors, *Asaia*, *Pantoea*, *Serratia*, *Pseudomonas* and *Thorsellia* have been evaluated as candidates for paratransgenesis [206,207,208].

Proof-of-principle experiments conducted with *An. gambiae* and *An. stephensis* [209,210,211] suggest paratransgenesis can be developed in an actual tool for malaria control. Engineered *Pantoea agglomerans* expressing anti-plasmodial genes inhibit the development of malaria parasites by up to 98% and reduce the proportion of infected mosquitoes by 84% in lab settings [210]. Similar results were obtained with *Serratia,* strain AS1 [209]. With this in mind, the microbial flora associated with *An. darlingi* and other neotropical malaria vectors have been investigated [197,212,213,214,215,216,217,218,219,220,221]. Expanding the knowledge of culturable bacteria associated with this malaria vector and identifying symbiotic bacterial strains that are amenable to genetic manipulation, colonize *An. darlingi* efficiently and are transferred from adult females to their progeny, is essential for moving forward and testing the viability of paratransgenesis for malaria control in the Amazon. Recent detection of *Asaia sp* in *An. darlingi* further supports the prospect of using these bacteria as tools for malaria control through paratransgenesis in the Amazon [212,218] although several challenges remain to be addressed for field applications (i.e., effectiveness, safety and release methods).

#### 5.2.4. Wolbachia

*Wolbachia* are common intracellular endosymbiont bacteria present in up to 60% of insect species, including mosquitoes [222,223,224]. They are maternally inherited and can cause reproductive alterations in their hosts, including cytoplasmic incompatibility (CI), parthenogenesis, male-killing, and feminization [223,225,226,227]. Furthermore, *Wolbachia* can inhibit the replication of pathogens in its arthropod hosts, making these organisms a promising tool to combat mosquito-transmitted diseases [228,229]. The successes of *Wolbachia*-based biocontrol of dengue and other arboviruses [230] suggest the possibility of similar *Wolbachia*-based strategies for malaria control. Additionally, applications of *Wolbachia* in combined incompatible and sterile insect technique (IIT-SIT) have been considered an effective and safe approach to control mosquito populations [231].

Evidences of natural *Wolbachia* infections in malaria vectors [232,233,234] triggered investigations on the possible use of *Wolbachia*–*Anopheles* associations to limit malaria transmission. These efforts generated remarkable results showing reduced egg laying (population reduction) and a significantly reduced *Plasmodium* prevalence in mosquitoes carrying native *Wolbachia* infection (population replacement) [233,235]. However, challenges remain for naturally occurring *Wolbachia* to be applicable as tools for malaria control. These strategies must rely on CI for *Wolbachia* to spread in natural populations and at present, it is not clear whether native *Wolbachia* can induce CI in anophelines. Induction of CI was not observed in caged experiments using wAnga-BF-infected *An. coluzzii* [235]. Nonetheless, *Wolbachia*-based malaria control strategies, such as population suppression or blocking of parasite development, are not only reliant on *Wolbachia* symbionts naturally associated with a given mosquito species. Successful dengue control was achieved with *Aedes aegypti* mosquitoes artificially infected with *Wolbachia* from a different insect species. Hence, the *Wolbachia*-based vector population suppression and disease transmission blocking can work in species not commonly infected with *Wolbachia* in the wild [236]. So far, the only *Anopheles* species amenable to *Wolbachia* transinfection in the laboratory is *An. stephensi* [237]. The wAlbB strain was used to stably infect *An. stephensi*, inducing complete CI, and conferring resistance to malaria parasites [238]. Recent studies suggest paratransgenesis could be exploited to circumvent difficulties in infecting malaria vectors with living *Wolbachia* strains. *Wolbachia*-derived molecules that stimulate the mosquito immune system and modulate vector competence, could be expressed in engineered symbiotic bacteria affecting parasite development [239].

*Wolbachia*-based approaches for malaria control in the Amazon have not been investigated to date. Successful laboratory colonization of *An. darlingi* and other local malaria vectors [162,163,164], will allow attempts to transinfect these mosquitoes with *Wolbachia*. However, *Wolbachia*-based approaches for malaria control in the Amazon will depend on vertical transmission of *Wolbachia* to offspring. Additional research is needed to investigate if CI, parthenogenesis or feminization could be induced by *Wolbachia* infection and to identify *Wolbachia* strains that affect malaria parasites development in *An. darlingi* and other neotropical anopheline mosquitoes. 

## 6. Final Remarks and Perspectives

Despite the WHO’s global efforts to control and eliminate malaria, the present malaria situation is still alarming, with an estimated 228 million yearly cases of malaria occurring worldwide, causing more than 400,000 deaths and predominantly affecting the poor and underprivileged [8]. While most malaria cases and related deaths occur in the World Health Organization (WHO) African Region (213 million or 93%), in 2018 the Americas reported more than 750,000 confirmed malaria cases, with 130 million people living in areas at risk of malaria transmission. Approximately 200,000 malaria cases were registered in the Brazilian Amazon in 2018. With the goal of providing the deserved health care to the thousands of people living in the Amazon, and in accordance with the United Nations Sustainable Development Goal (SDG) 3, “ensure healthy lives and promote well-being for all at all ages” [240], increased investment and adequate planning will be necessary to eliminate malaria transmission in the area. Investments in malaria elimination and eradication are worthwhile, resulting in millions of lives saved, stimulating the economy and fostering prosperity, ensuring return on investment of billions of dollars [241,242,243,244,245,246]. 

While malaria elimination in Brazil in the near future remains unlikely [7], researchers are exploring and developing novel and promising vector-based approaches to curb malaria transmission. Along with improvements of vaccines, drugs, diagnostic tools, IRS, and insecticide-treated nets, new vector-based approaches may prove crucial for the implementation of malaria-control programs, especially in regions where existing interventions have been unable to eliminate disease transmission. The efficacy and biosafety concerning these new technologies will need to be addressed via a stepwise regulatory framework before they can be incorporated into malaria control programs. Meanwhile, research expanding the knowledge of neotropical malaria vectors’ biology, ecology, behavior, physiology, genetics, biochemistry, and insecticide resistance, primarily with regards to *An. darlingi*, is needed, as it is the basis on which vector-based malaria control in the Amazon may be founded.

## Figures and Tables

**Table 1:** Current Guidelines for Malaria Vector Control in the World and in Brazil.

World Health Organization	Brazilian Ministry of Health
GUIDELINES FOR MALARIA VECTOR CONTROL 2019 [83] Malaria vector control, Policy guidance, Recommendations, accessed on May 5, 2020 [84] Malaria vector control, Policy guidance, Operational manuals, accessed on May 5, 2020 [85]	Malária: o que é, causas, sintomas, tratamento, diagnóstico e prevenção, accessed on May 5, 2020 [14] Plano de Eliminação da Malária no Brasil 2016 [6] Guia de tratamento da malária no Brasil 2020 [86]
**Core interventions**	
INSECTICIDE-TREATED NETS: Pyrethroid-only Long-lasting insecticidal nets (LLINs) prequalified by WHO are recommended for deployment as a core intervention in all malaria-endemic settings. Pyrethroid-PBO nets prequalified by WHO are conditionally recommended for deployment instead of pyrethroid-only LLINs where the principal malaria vector(s) exhibit pyrethroid resistance. Strongly recommended as an intervention of public health value, high-certainty evidence.	INSECTICIDE-TREATED NETS: Use of long-lasting impregnated mosquito nets in priority locations for each municipality and increase coverage in locations where LLIN is already used, together with monitoring of LLIN replacement plan to ensure availability.
INDOOR RESIDUAL SPRAYING (IRS): IRS deploying a product prequalified by WHO is recommended as a core intervention in all malaria-endemic settings. DDT has not been prequalified; it may be used for IRS if no equally effective and efficient alternative is available, and if it is used in line with the Stockholm Convention on Persistent Organic Pollutants. Strongly recommended as an intervention of public health value, low-certainty evidence.	INDOOR RESIDUAL SPRAYING: IRS following technical recommendations of the Brazilian Health Surveillance Secretariat (SVS), in buildings located in areas responsible for 80% of malaria transmission by Infection, and in cycles that allow residual insecticide to be maintained throughout the year.
	HOUSING AND WORKING PLACE IMPROVEMENT: doors and windows screen installation and maintenance.
**Supplementary interventions**	
LARVICIDING: Regular application of biological or chemical insecticides to water bodies is recommended in areas where high coverage with a core intervention has been achieved, where aquatic habitats of the principal malaria vector(s) are few, fixed and findable, and where its application is both feasible and cost-effective. Conditionally recommended as an intervention of public health value, low-certainty evidence.	LARVICIDING: Carrying out management of water collections to eliminate breeding sites of anopheline mosquitoes in urban locations with malaria transmission. Drainage; minor sanitation work to eliminate vector breeding sites; landfill; cleaning the margins of breeding sites; modification of water flow; control of aquatic vegetation.
**Personal protection measures**	
TOPICAL REPELLENTS: Deployment of topical repellents is not recommended as a public health intervention; however, topical repellents may be beneficial as an intervention to provide personal protection. Conditionally recommended against deployment as an intervention with public health value, low-certainty evidence.	TOPICAL REPELLENTS: DEET (N-N-dietilmetatoluamida)
INSECTICIDE-TREATED CLOTHING: Use of insecticide-treated clothing is not recommended as an intervention with public health value; however, insecticide-treated clothing may be beneficial as an intervention to provide personal protection in specific population groups. Conditionally recommended against deployment as an intervention with public health value, low-certainty evidence.	CLOTHING: Clothing that protects legs (pants) and arms (long sleeve)
SPACE SPRAYING: Space spraying should not be undertaken for malaria control, and IRS or LLINs should be prioritized instead. Conditionally recommended against deployment, very low-certainty evidence.	SPACE SPRAYING: Performing chemical spatial control, when in outbreak situations.
